# Bioassay-Guided Fractionation, ESI-MS Scan, Phytochemical Screening, and Antiplasmodial Activity of *Afzelia africana*

**DOI:** 10.1155/2022/6895560

**Published:** 2022-04-13

**Authors:** Bright Yaw Vigbedor, Jonathan Osei-Owusu, Ralph Kwakye, David Neglo

**Affiliations:** ^1^Department of Basic Sciences, School of Basic and Biomedical Sciences, University of Health and Allied Sciences, PMB31, Ho, Ghana; ^2^Department of Biological, Physical and Mathematical Sciences, University of Environment and Sustainable Development, PMB, Somanya, Ghana

## Abstract

**Background:**

*Afzelia africana* is a plant species with reported numerous medicinal potentials and secondary metabolites. Various parts of the plant have been applied for the treatment of hernia, rheumatism, pain, lumbago, malaria, etc. The study seeks to evaluate the phytochemical constituents, antiplasmodial, and ESI-MS scan of bioassay-guided fractions from the methanol extract of the bark of the plant.

**Aims:**

The main aim of the study was to carry out bioassay-guided fractionation of the crude methanol extract of *Afzelia africana* in order to isolate fractions and to evaluate their antiplasmodial activities and ESI-MS fingerprints.

**Methods:**

The methods employed include column chromatographic fractionation, phytochemical screening, antiplasmodial activity (malaria SYBER green assay (MSF)), and ESI-MS profile (full ESI-MS scan).

**Results:**

The column chromatographic fractionation and phytochemical screening of the plant led to the separation of the following four fractions: **1** (flavonoids, phenolics, glycosides, terpenoids, and steroids), **2** (alkaloids, anthraquinones, flavonoids, phenolics, glycosides, terpenoids, and steroids), **3** (anthraquinones, flavonoids, phenolics, glycosides, terpenoids, and steroids), and **4** (alkaloids, flavonoids, phenolics, glycosides, terpenoids, and steroids). The antiplasmodial activities of the fractions were tested against the 3D7 strain of *Plasmodium falciparum* with reported stronger activities for **1** (IC_50_: 0.097 ± 0.034 *μ*g/mL) and **3** (IC_50_: 1.43 ± 0.072 *μ*g/mL), and weaker activities for **2** (IC_50_: >100 *μ*g/mL) and **4** (IC_50_: 37.09 ± 6.14 *μ*g/mL). The full ESI-MS fingerprint of fractions **1**, **2**, **3**, and **4** revealed the presence of 14, 24, 34, and 37 major molecular ions or compounds in each fraction, respectively.

## 1. Introduction

Malaria is a parasitic disease transmitted by the female anopheles mosquito through the injection of the *Plasmodium falciparum* into the bloodstream of the host organism [1–3]. Statistical data from the WHO indicated a total of 229 and 228 million cases for 2018 and 2019, respectively. The number of deaths recorded within the duration stood at 411,000 and 409,000, respectively [4, 5]. The infant-reported death cases accounted for about 67% (274,000) of the 2019 deaths. In view of the higher infant mortality, the WHO further recommended the use of the RTS, SAS01 malaria vaccine for children [4, 5]. Previous intervention in the treatment of malaria involved single and combination therapy with known antiplasmodial compounds such as the derivatives of aminoquinoline and artesunate. However, antimalarial drug resistance has emerged as one of the challenges facing malaria treatment [4–6].

The biggest intervention in the chemical treatment regime for malaria has derived its source from herbal medicines [7]. The discovery of quinine and artesunate from natural sources rekindled giant steps into a rigorous search for secondary metabolites from plant sources. The utilization and derivation of treatment regimes from herbal medicines have gone through a two-way approach, that is, a rigorous approach through bioassay-guided fractionation to identify pure compounds and the evaluation of its antiplasmodial activity [8, 9]. The second approach has gone through the formulation of herbal medicines directly from extracts from the plants after the evaluation of its biological activity. Furthermore, standard parameters are applied to standardize it for human patronage [10–12].

In the area of antimalarial drug discovery, where the contribution of ethnopharmacology has been well documented includes the discovery of quinine from the quincona plant, artemisinin from *Artemisinin annua*, and the isolation of cryptolepis from the West African plant, *Cryptolepis sanguinolenta* [13]. Over the last 20 years, 175 plant-derived antiplasmodial compounds have been published with several of them showing a nanomolar (nM) range of activity [14]. It is therefore evident that natural products from plants are endowed with thousands of secondary metabolites; however, the exact composition and the interactive effects of these secondary metabolites could be optimized to obtain them in different compositions [15]. Bioassay-guided isolation of bioactive compounds is a modern and efficient technique in metabolites screening. Through bioassay-guided fractionation studies, secondary metabolites have been obtained in fractions containing compounds existing in different compositions in several mixtures. Such processes have yielded fractions that have generated biological activities not originally present in the crude extracts [16]. Currently, advanced analytical tools and techniques have helped to further fractionate, isolate, and purify compounds from plants, and their biological activities are evaluated. Analytical techniques that have been employed include chromatographic techniques (LC, HPLC, and TLC) and spectra techniques (mass spectrometry, infrared, ultraviolet/visible, nuclear magnetic resonance, X-ray) [17, 18].

*Afzelia africana* is a plant species belonging to the Fabaceae family. The plant is commonly referred to as African mahogany and thrives well in the tropical regions of Africa. Various parts of the plant have been applied in traditional medicines such as analgesic, emetic, laxative, febrifuge, hemorrhagic, malaria, rheumatism, diuretic, paralysis, and lumbago. Furthermore, the pulverized mixture of the roots with millet beer is used to treat hernias and, whilst a decoction with pimento, is used as a remedy against gonorrhea and stomach ache. A leaf decoction, combined with *Syzygium guineensis* leaves and Xylopia fruit, forms a drink that is used to treat oedema [19–22]. The various reported ethnobotanical applications of the plant that have been authenticated by laboratory investigations include anthelmintic [23], antioxidant [24], anti-inflammatory/analgesic [25], and antitrypanocidal [26, 27]. Furthermore, the ethnobotanical utilization of the plant as antiplasmodial agent has been confirmed against the 3D7 strain of *Plasmodium falciparum* (IC_50_: 2.97 *μ*g/mL) [28]. The antimicrobial [29, 30], antidiabetic, liver, and kidney protective ability (against alanine transaminase (ALT) and aspartate aminotransferase (AST), bilirubin, urea, and creatinine) are further reported with significant outcomes [31, 32]. In view of the vast medicinal properties reported for the plant, further studies seek to carry out an extensive bioassay-guided fraction of the methanol extract and the evaluation of its phytochemical and antiplasmodial activities. In this study, the ESI scan of the various fractions will be investigated to give a fair insight into the phytochemical constituents, number of compounds, or molecular ions present in them.

## 2. Materials and Methods

### 2.1. Materials

#### 2.1.1. Plant Materials

The Bark of *Afzelia africana* was obtained from the Sekyere Central District of the Ashanti Region. The plant material was further identified by Mr. Clifford Osafo Asare at the Department of Herbal Medicine, Faculty of Pharmacy and Pharmaceutical Sciences, Kwame Nkrumah University of Science and Technology, Kumasi, Ghana. A voucher specimen with voucher number [KNUST/HM1/2019/SB/008] was deposited in the Herbarium Unit for reference purposes.

#### 2.1.2. Test Organism

The test organism (3D7 strain of *Plasmodium falciparum*) was obtained from the Department of Chemical Pathology, Noguchi Memorial Institute for Medical Research, University of Ghana, Legon.

#### 2.1.3. Reagents

Artesunate (98%) was purchased from Sigma-Aldrich (St. Louis, MO, USA). Gentamicin was obtained from Invitrogen Life Technologies Inc. (Carlsbad, CA, USA). RPMI-1640 medium, streptomycin/penicillin, L-glutamine, and HEPES were obtained from Gibco BRL Life Technologies (Grand Island, NY, USA).

### 2.2. Extraction of Plant Material

A 500 g of the bark was dried under shade for three (3) weeks to reduce the moisture content. The dried plant material was further pulverized and then cold macerated in 90% methanol. The mixture was then stored for 72 hours with occasional swirling and shaking. The extract was then filtered by a vacuum pump to obtain the filtrate. The filtration procedure was repeated two more times with fresh 90% methanol solution until enough filtrate was obtained. The filtrate was further concentrated using the rotavap and then dried to obtain a solid residue. The percentage yield of the successive extractions was 16% (80) g. The solid crude extract was stored at 2°C until further use.

### 2.3. Column Chromatography and Bioassay-Guided Fractionation

Sixty grams (60 g) of the methanol extract was mixed with 100 g of silica gel 60 (0.05–0.2 mm (230–400 mesh)) in 100 mL methanol and then dried under reduced pressure. The adsorbed extract was then mounted on a column (open, 45 × 4.5 cm) containing 250 g silica gel (230–400 mesh size). The mounted column was eluted with the mobile phase solvent system comprising of petroleum ether, petroleum ether–ethyl acetate, and ethyl acetate through gradient elution. The procedure yielded 40 fractions based on TLC profiles using a solvent system of pet ether/ethyl acetate (1 : 1). Spots were visualized by using saturated iodine vapour and anisaldehyde solution. The fractions were further pooled into four fractions based on thin-layer chromatography (TLC) profile to give **1 (**200 mg, pale yellow powder), **2** (brown precipitate, 342 mg), **3 (**300 mg, yellow powder), and **4** (500 mg, brown precipitate).

### 2.4. Phytochemical Screening of Fractions

The phytochemical investigations of the fractions were achieved by adopting the procedure by Amir et al. [33] as described in the experimental procedures. The phytochemical classes that were tested for the fractions include alkaloids, flavonoids, saponins, coumarins, anthraquinones, steroids, and glycosides.

### 2.5. Antiplasmodial Screening of Fractions

Antimalarial activities of the fractions were investigated by adopting the malaria SYBER green fluorescence (MSF) assay method by Kwansa-Bentum et al. [34] with slight modification. A highly synchronous ring-stage parasite was used in the assay.

#### 2.5.1. *In-Vitro* Cultivation of Malaria Parasite

Blood stages of the laboratory clones of chloroquine-sensitive 3D7 strain of *P. falciparum* were cultured *in-vitro* according to the method of Trager and Jensens. All culture steps were performed using the aseptic technique in the NuAire laminar flow class II safety cabinet. All glassware was autoclaved at 121°C (15 atmospheres) for at least 15 min. Malaria parasite was maintained in continuous culture with human packed red blood cells (blood group O^+^) in RPMI 1640 medium supplemented with 10% human AB^+^ serum, 25 mM N-2-hydroxyethylpiperazine-N-2-ethanesulfonic acid (HEPES), 25 mM sodium bicarbonate, and gentamicin sulfate (60 *μ*g/ml, pH 7.2). The culture was incubated at 37°C in an atmosphere consisting of 90% N_2_, 5% O_2_, and 5%. Parasite culture was synchronized to the ring stage by treatment with 5% (w/v) D-sorbitol.

#### 2.5.2. Assessment of *In Vitro* Antimalarial Activity of Fractions

An aliquot of parasite inoculum (50 *μ*L) with 0.5% parasitaemia and 2% haematocrit was added into each well of the microtiter plate. The fraction dissolved in DMSO and diluted with RPMI 1640 to a final concentration of 1% was added to the malaria culture at six final concentrations of 100, 25, 6.25, 1.56, 0.39, and 0.098 *μ*g/mL with artesunate (ART) as a standard antimalarial drug. Wells without the fractions but the culture at the same parasitaemia and haematocrit in distilled water were included on the 96-well plate as control. Using the candle jar method, the plate was put into a humidified chamber in an incubator at 37°C for 72 hours. The antiplasmodial activities of the fractions were deduced by the malaria SYBR green fluorescence (MSF) assay. After 72 hours of incubation, 100 *μ*L of the SYBR green 1 fluorescence lysis buffer containing 20 mM Tris-Cl (pH 7.5), 5 mM EDTA, 0.008% saponin, and 0.08% triton-X 100 and SBR green were added to each prepared well. The plate was further covered with aluminum foil and then incubated in the dark at room temperature for 3 hours. The fluorescence generated by each well was read on the multiwell plate reader (Tecan Infinite M200). The experiment was repeated two more times (triplicate each). The evaluation of the percentage inhibition was done by inputting the parasitaemia of control and the parasitaemia of serially diluted concentrations of test samples into the relation.

(1) $$\begin{array}{c} \%\text{ Inhibition}=\left[\frac{\text{Parasitaemia of control}-\text{Parasitaemia of test fraction}}{\text{Parasitaemia of control}}\right]\times 100\%. \end{array}$$

The % inhibition versus the logarithm of the concentrations [LogC] of each fraction was plotted. The IC_50_ (*μ*g/mL) of the fractions were further determined by employing the GraphPad Prism software package 6 (version 6.01) (San Diego, California USA) with non-regression (curve fit) using the log(inhibitor) vs response (three parameters).

### 2.6. Full Scan ESI-Ms Fingerprint of Fractions

The ESI-MS fingerprint of the fractions was carried out using the Quantum Access MAX mass spectrometer equipped with an ESI source. Both the negative and the positive ion mode were applied with a source voltage of 100 V, the capillary temperature of 250°C, the nebulizing gas flow rate of 1.5 mL/min, and collision energy of 100 V. The spectra data were obtained with full-scan mode for m/z in the range of 100 to 700. The solvent system for elution comprises of an isocratic volume of methanol/water, 50 : 50.

### 2.7. Statistical Analysis of Data

The dose-response curves from the experimental data were analyzed by using the Graphpad Prism software package 6 for Windows (San Diego, California USA). The data were further analyzed by the one-way ANOVA using GraphPad Prism 6 software with a significant difference at *P* < 0.01. The data from the experiment were reported as mean and SD values from triplicate measurements.

## 3. Results and Discussion

The principle of bioassay-guided fractionation has played a significant role in understanding the phytochemistry, pharmacognosy, and pharmacology of herbal medicines [15]. The principle has further led to the linkage of various fractions from plants to their biological activity and specific compounds directly linked to their biological activities [35]. In the current study, the principle of column chromatographic fractionation was employed to obtain fractions that were bulked together based on a similar retention factor (*R*_*f*_). The crude methanol extract of the *A. africana* was obtained by employing the cold maceration technique with an overall yield of 16%. The crude extract was subsequently mounted on the column and eluted with a solvent system comprising petroleum ether and ethyl acetate. The procedure led to the isolation of four fractions. Fraction **1** (comprising three spots on TLC (*R*_*f*_: 0.18, 0.36, 0.55)) was obtained as a yellow isolate with a solvent system of pet ether/ethyl acetate (85 : 15). Fractions **2**, **3**, and **4** were obtained with solvent systems of pet ether/ethyl acetate (75 : 25), pet ether/ethyl acetate (70 : 30), and pet ether/ethyl acetate (70 : 30), respectively. The fractions were bulked together based on the TLC profile (number of spots and their retentions times) and represented in Table 1.

The biological activity and, specifically, the antiplasmodial activity of plants have been linked to the presence of secondary metabolites and their synergistic effects [36, 37]. The existence of two or more secondary metabolites in their right compositions and permutations could be harnessed and optimized to yield resultant beneficial outcomes, notwithstanding, their associated toxic and adverse effects [36]. Phytochemical screening of secondary metabolites from plants is the first step in understanding the phytochemistry, pharmacognosy, and pharmacology of extracts from plants [15]. The phytochemical screening provides a rich source of information about the composition and structural varieties of compounds in extracts from plants [39]. Nevertheless, the test could further provide clues into biological activities, toxicities, and structures of active compounds for authenticating the ethnobotanical utilization of plant medicines [15]. As a result, well-standardized methods for screening alkaloids, terpenoids, steroids, flavonoids, phenolics, tannins, coumarins, anthraquinones are well documented [36]. The application of a standardized procedure for the screening of the phytochemical constituent of the plant is indicated in Table 2.

**Figure 1:**
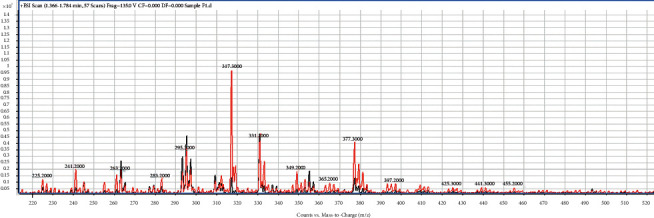
ESI/MS full scan of fraction **1**.

**Figure 2:**
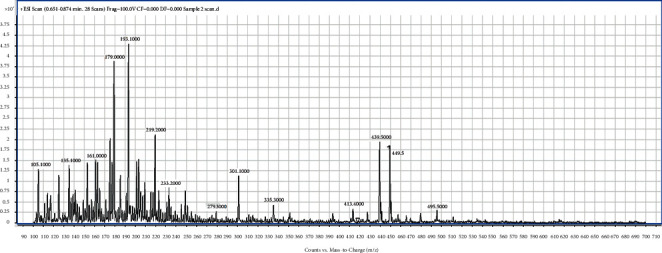
ESI/MS full scan of fraction **2**.

**Figure 3:**
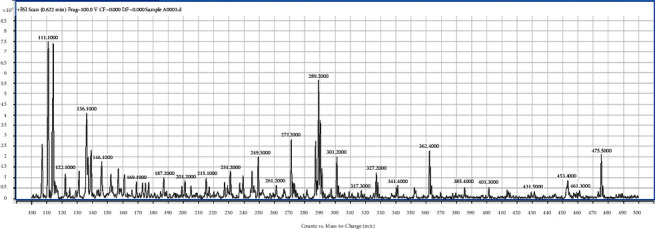
ESI/MS full scan of fraction **3**.

**Figure 4:**
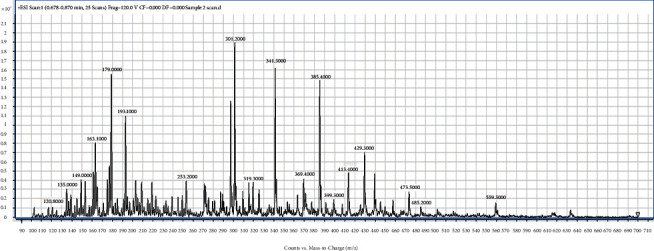
ESI/MS full scan of fraction **4**.

From Table 2, the isolated fractions indicated the presence of several secondary metabolites for **1** (flavonoids, phenolics, glycosides, terpenoids, and steroids), **2** (alkaloids, anthraquinones, flavonoids, phenolics, glycosides, terpenoids, and steroids), **3** (anthraquinones, flavonoids, phenolics, glycosides, terpenoids, and steroids), and **4** (alkaloids, flavonoids, phenolics, glycosides, terpenoids, and steroids). The phytochemical screening results have further revealed the presence of wide varieties of secondary metabolites present in the fractions from the bark of *Afzelia africana*. The presence of these secondary metabolites could account for the numerous medicinal properties reported for *Afzelia africana*. The phytochemical screening experiment indicating the presence of the phytochemicals in *Afzelia africana* further confirms similar results determined by other studies [31, 40, 41]. Further experimental procedures to justify the presence of the wide varieties of secondary metabolites in the four fractions were achieved by performing a full ESI-MS scan of the fractions. The single-stage full ESI-MS scanning of bioactive compounds in crude extracts from medicinal plants has attracted widespread interest and promoted a much better understanding of traditional herb medicines in recent years. Currently, liquid chromatography coupled with electrospray ionization (ESI) tandem mass spectrometry (LC-ESI-MS) has been widely used for the fast separation and structural identification of secondary metabolites without tedious isolation and purification of pure compounds from the complex sample matrix. Furthermore, several useful information is obtained from the MS experiment by MS full scan [39, 42]. The full ESI-MS scans of fractions **1**, **2**, **3**, and **4** are displayed in Figures 1–4.

**Figure 5:**
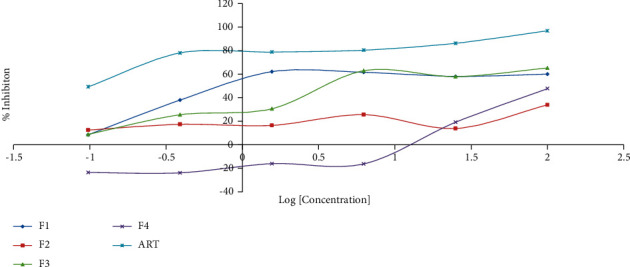
A plot of % inhibition of fractions versus log of concentration.

The ESI-MS scan figures revealed the identification of 14, 24, 34, and 37 major molecular ions in fractions **1** (225.2, 241.2, 263.8, 283.2, 295.8, 317.3, 331.1, 349.2, 365.2, 377.3, 397.2, 425.3, 441.3, 455.2), **2** (105.1, 181.1, 119.1, 125.1, 135.1, 161.0, 178.1, 179.1, 185.1, 193.1, 201.1, 209.1, 216.1, 219.2, 221.1, 233.2, 249.1, 279.3, 301.1, 335.3, 413.4, 439.5, 449.5, 495.5), **3** (97.1, 111.1, 114.1, 122.1, 131.1, 136.1, 139.1, 146.1, 153.1, 158.1, 169.1, 175.1, 177.1, 178.1, 187.2, 201.2, 215.1, 231.2, 239.1, 247.1, 249.3, 261.2, 271.2, 289.2, 301.2, 327.2, 341.4, 362.4, 385.4, 401.3, 431.5, 453.4, 461.3, 475.5), and **4** (120.8, 135.0, 149.0, 152.0, 163.1, 179.0, 193.1, 205.1, 210.1, 215.1, 220.1, 235.1, 239.1, 245.1, 249.1, 253.2, 271.1, 280.1, 289.1, 299.1, 301.2, 304.1, 319.3, 327.0, 341.3, 369.4, 385.4, 399.3, 413.3, 429.3, 439.5, 445.1, 450.1, 458.5, 473.5, 485.2, 559.3), respectively. The results indicate the presence of wide varieties of molecular ion in the fractions and further confirming the phytochemical screening results (Table 3). In view of the presence of the wide varieties of secondary metabolites in the isolated fractions, they were submitted to *in-vitro* antiplasmodial testing against *P. falciparum*, 3D7 strain. A parasite culture was carried out as already described by Kwansa Bentum et al. [34]. The fractions were tested in triplicate, and the results are expressed as mean ± SD. The antiplasmodial activities of the isolated fractions were tested against 3D7 strain of *Plasmodium falciparum* through the malaria SYBER green fluorescence (MSF) assay method. The concentration range for the percentage inhibition assay was within the range of 100–0.098 *μ*g/mL. The results for the plot of the percentage inhibition against the corresponding logarithm of the concentrations for the fractions and their IC_50_ (*s*) are shown in Figure 5 and Table 3.

The classification of the activity of the fractions was achieved by adopting the criteria, where IC_50_ < 5 *μ*g/mL was classified as very active; promising activity between 5 and 15 *μ*g/mL; moderate activity between 15 and 50 *μ*g/mL; and inactive at IC_50_ > 50 *μ*g/mL [43–45]. From the graph, the four fractions exhibited inhibitory activities for the various concentrations. For instance, fractions **1** and **3** exhibited stronger activity with percentage inhibitions within the range of (60–8)% and (65–8)% for the various concentrations. The fifty percentage inhibitory concentrations (IC_50_) for the fractions reported values of 0.097 ± 0.034 and 1.43 ± 0.072 *μ*g/mL for **1** and **3**, respectively. Preliminary phytochemical screening for **1** and **3** indicated the presence of phenolics, steroids, coumarins, alkaloids, and flavonoids, and these could account for the stronger activities. The observation further corroborates the antiplasmodial activity reported for the crude methanol extracts with an IC_50_ value of 2.97 *μ*g/mL [28] and further supports the use of this plant in traditional medicine [46]. The fractions **2** and **4**, however, respectively, recorded inactive and moderate inhibitory activities for the concentrations with inhibitory concentrations within the ranges of (up to 33) and (up to 47)%. The IC_50_ values for **2** and **4** were found to be greater than 100 *μ*g/mL for fraction **2** and 37.09 ± 6.14 *μ*g/mL for fraction **4**. The negative inhibitory values of the percentage inhibitions for the fraction F4 could be due to the proliferation of the plasmodium species enhanced by the fractions. Several such observations are reported, and at specific concentrations, they achieve proliferation, and in other instances, they act as apoptosis [47]. In such instances, they significantly inhibit cell proliferation only at high concentrations but increase apoptosis at low concentrations. These data demonstrate that the effects of the fraction are concentration-dependent [47, 48]. Several classes of phytochemicals with such reported properties include resveratrol, epigallocatechin gallate, gingerol, phytosterol, and myricetin. They are known to directly influence various molecular signal transduction pathways such as inflammation cascade, cell proliferation/migration, oxidative stress, and metabolic disorders, which are involved in the development of several noncommunicable diseases [49]. Hence, the presence of a similar class of compounds in the fractions could contribute to the observation. The fractions could therefore be found to support accelerated cell divisions resulting in exponential cell proliferation faster than the positive control. For instance, several phytochemical classes such as triterpenoids, alkaloids, coumarins, flavonoids, steroids have been reported to contribute to the antiplasmodial activity of extracts [37, 50, 49]. Furthermore, *Afzelia africana* belongs to the Fabaceae family which has been known to exhibit promising antiplasmodial activities, with several active compounds isolated from them [52, 53].

Furthermore, the activities of the fractions (**2** and **4**) were relatively weaker, in spite of the rich phytochemical constituents detected in them. The observation could be due to the activity of constituents being masked by other compounds when in combination. Such phenomenon is reported, where the coexistence of two or more secondary metabolites could either produce competitive or noncompetitive antagonism [54]. Furthermore, secondary metabolites present in plants are classified into various phytochemical classes such as phenolic, flavonoid, and terpenoids. However, a particular phytochemical class has vast structural diversities with different physicochemical properties (hydrophilicity, lipophilicity, concentration, pH value, solubility, and bioavailability in critical biological targets) that could enhance or reduce their respective biological activities [55]. Therefore, the fact that the extracts have the same phytochemical composition or class does not mean they will register the same level of activity [55]. As a result, the current weaker activity cannot justify the rejection of the activities of the individual compounds in the fractions. It is therefore a good practice to carry out bioassay-guided fractionation in order to identify active fractions that could individually serve as drugs or in their combined state. Nevertheless, the active fractions could further be subfractionated in order to identify active compounds that could further serve as drugs or lead agents for further studies. The myriad of chemical (phytochemical, ESI-MS) and biological (antiplasmodial) data reported for the plant could therefore account for the ethnobotanical utilization of *A. africana* for the treatment of malaria. However, to account for the ethnobotanical utilization of *A. africana* as an antimalarial remedy as well as the myriad of promising antiplasmodial activity reported, a review of the mechanism of action of the phytochemical classes identified in the plant could provide further insight. Several literature suggests that the mechanism of action of most phytochemicals is dependent largely on the site of action. Moreover, phytochemicals have been known to exhibit their mechanism of biological activity by interfering with a biosynthetic pathway, cell wall destruction, DNA intercalation, interference with DNA transcription, and protein synthesis [56, 57]. Furthermore, in order to account for the exact mechanism of action and the contribution of phytochemicals to the antiplasmodial activity of plants, the mechanism of action and drug-resistant activity of *P. falciparum* must be taken into account. Over the years, *P. falciparum* has developed several mechanisms for survival in the host organism as well as resisting drug activity, that is, drug resistance [58]. The parasite after infecting the host organism and degrading the RBC for its protein synthesis and other life processes (life stages: trophozoites, merozoites, and the schizonts) has been known to utilize the conversion of heme (Fe(II) protoporphyrin (IX): a hemoglobin metabolite) to hemozoin (a protective polymer) as a means of sequestering the toxic effect of the former (heme) [59]. Hence, targeting this mode of action involves the screening of compounds that could inhibit the synthesis of hemozoin in the parasite which subsequently leads to the accumulation of the toxic heme, leading to cytotoxicity and cell death. Phytochemicals with such inhibitory activity against heme-hemozoin conversion include alkaloids classes such as quinine, quinidine, cinchonine, and cinchonidine [60, 61]. Furthermore, the antiplasmodial activities of several anthraquinones, phenolics, and flavonoids could be linked to their ability to form *π*-*π* stacking interaction with heme leading to binding and subsequent inhibition of hemozoin formation in the parasite [62–64]. The major pharmacophore responsible for the activity of these phytocompounds is due to the presence of multiple phenolic systems in their structures. Specific phytocompounds identified to follow this mechanism of action include rufigallol, exifone, apigenin, ellagic acid, and derivatives [65–67]. The existence of alkaloids, flavonoids, phenolics, anthraquinones as well as other phytochemicals in the fractions could therefore account for their antiplasmodial activities.

In other studies, *P. falciparum* have been discovered to utilize glutathione for its inhibitory effect against drug activity. The glutathione-s-transferase enzyme (GST) has been a key enzyme in the biosynthesis of glutathione. In fact, the enzyme represents 1–10% of the cellular proteins of *Plasmodium falciparum* [68]. The enzyme has been implicated in the antimalarial drug-resistant capacity of *P. falciparum*. Hence, phytocompounds with inhibitory activity against the enzyme could provide a route for addressing drug resistance. Several classes of flavonoids and polyphenolic compounds isolated from plants have been found to inhibit the action of glutathione-s-transferase such as derivatives of the compounds of ellagic acid, quercetin, rutin, kaempferol, purpurogallin due to the presence of phenolic groups [69–71]. It is experimentally documented that flavonoids or compounds with *α*-*β* unsaturated carbonyl groups and polyphenolic systems form monoadduct with L-cysteine of the enzyme (glutathione-s-transferase) through their *α*-*β*-unsaturated carbonyl moiety via the Michael addition mechanism [69–71]. The resultant effect could therefore lead to the inhibition of the protective capacity of the enzyme, leading to cytotoxicity and cell death [72]. As a result, the presence of flavonoids and phenolic compounds with similar structural motifs in fractions **1**, **2**, **3**, and **4** (Table 2) could therefore account for their reported antiplasmodial activity.

Other mechanisms of antimalarial activity of phytocompounds have been linked to their ability to antagonize folic acid's primary function. It is established that folic acid is involved in the biosynthesis of key biomolecules such as amino acids (e.g., serine, methionine) and DNA/RNA components (e.g., thymidine and purine bases) [73]. Antiplasmodial compounds with the antifolate mechanism inhibit folic acid synthesis by antagonizing the main enzyme, dihydrofolate reductase (DHFR), which is involved in the folic acid synthesis. The inhibition of the enzyme further produces a series of cascading effects such as the impairment of DNA, RNA, and protein synthesis in *P. falciparum* leading to cell death [74]. Several phytochemicals such as alkaloids (piperine, berberine, dictamine reserpine), flavonoids (baicalein, biochanin, kaempferol, chalcone, catechin), coumarins, and terpenes have been found as potential folate synthesis inhibitors [56, 75, 76]. The phytochemical screening (Table 2) indicated the presence of a similar class of compounds in fractions **1**, **2**, **3**, and **4** and could further account for the reported antiplasmodial and ethnobotanical application of *A. africana* as a malarial remedy.

## 4. Conclusion

The bioassay-guided fractionation of the bark of *Afzelia africana* led to the isolation of four distinct fractions with varying degrees of antiplasmodial activities against the 3D7 strain of *P. falciparum*. The study further reveals the presence of secondary metabolites such as alkaloids, anthraquinones, flavonoids, phenolic, glycosides, terpenoids, steroids, terpenoids, and steroids. The results of the study confirm the ethnobotanical utilization of *A. africana* as an herbal treatment for malaria and other diseases.

## Figures and Tables

**Table 1:** TLC profile of fractions.

Pooled fraction	Colour	Solvent system	Method detection	No of spots	*R*_*f*_
**Fo** (1–19)		Pet ether	—	—	—
**1** ((20–21)	Pale yellow	Pet ether/ethyl acetate (85 : 15)	Anisaldehyde	3	0.18, 0.36, 0.55
**2** (22–30)	Brown	Pet ether/ethyl acetate (75 : 25)	Iodine vapour	6	0.76, 0.78, 0.84, 0.89, 0.93, 0.98
**3** (31–37)	Brown	Pet ether/ethyl acetate (70 : 30)	Iodine vapour	7	0.58, 0.67, 0.78, 0.84, 0.91, 0.96, 0.98
**4** (38–40)	Brown	Pet ether/ethyl acetate (70 : 30)	Iodine vapour	8	0.25, 0.55, 0.64, 0.71, 0.82, 0.85, 0.91, 0.95

**Table 2:** Phytochemical screening for fractions.

Phytochemical test	Fraction 1	Fraction 2	Fraction 3	Fraction 4
Alkaloids	−	+	−	+
Anthraquinone	−	+	+	−
Flavonoids	+	+	+	+
Phenolics	+	+	+	+
Coumarins	−	−	−	−
Glycosides	+	+	+	+
Terpenoids	+	+	+	+
Steroids	+	+	+	+

+: positive, −: negative.

**Table 3:** IC_50_ values for fractions.

Sample	IC_50_ (*μ*g/mL)	Comment
**1**	0.097 ± 0.034	Very active
**2**	>100	Inactive
**3**	1.43 ± 0.072	Very active
**4**	37.09 ± 6.14	Moderate
ART	0.03 ± 0.01	Very active

## Data Availability

The TLC profile (Table 1) data, the phytochemical screening (Table 2) data, the ESI/MS (Figures 1–4) data, the antiplasmodial activity (Figure 5) data, and the antiplasmodial activity IC50 (Table 3) data used to support the findings of this study are included within the article.
